# Survival of pancreatic cancer patients is negatively correlated with age at diagnosis: a population-based retrospective study

**DOI:** 10.1038/s41598-020-64068-3

**Published:** 2020-04-27

**Authors:** Hongcheng Wang, Jiazhe Liu, Guanggai Xia, Shizhou Lei, Xiuyan Huang, Xinyu Huang

**Affiliations:** 1 Department of General Surgery, Shanghai Jiao Tong University Affiliated Shanghai Sixth People’s Hospital, 600 Yishan Road, Shanghai, 200233 China; 2 Department of Hepatobiliary and Pancreatic Surgery, Minhang Branch, Zhongshan Hospital, Fudan University, Shanghai, China

**Keywords:** Outcomes research, Cancer epidemiology

## Abstract

In this population-based retrospective study, we aimed to investigate the association between age at diagnosis and prognosis of pancreatic cancer (PC) patients using data from the National Cancer Institute’s Surveillance, Epidemiology, and the End Results database. Different factors for stratification, like race, sex, year of diagnosis, pathological grade, American Joint Committee on Cancer stage, historic stage, and tumour location, were included to compare the survival rates of patients of different age groups, and the five-year survival rate was calculated. Multivariate analysis using Cox regression was performed to control for confounder bias, and the hazard ratio was calculated. In total, 126,066 patients were enrolled in this study. The five-year PC-specific survival of patients aged 20–40 years was almost three times that of patients aged >40 years. Stratified by race, sex, year of diagnosis, pathological grade, clinical stage, and tumour location, a descending trend of survival was observed with an increase in age. On multivariate analysis, the mortality risk of PC patients aged 40–80 years was twice that of patients aged <40 years; however, patients aged >80 years had a mortality risk three times that of patients aged <40 years. The survival rate of PC patients has improved in the last few decades. Age at diagnosis is a significant and negative prognostic factor for PC, and patients diagnosed at a relatively earlier stage had the best survival.

## Introduction

The incidence and mortality of pancreatic cancer (PC) are increasing with age in both sexes, and PC is mostly diagnosed in elderly individuals aged >70 years^1^. As a devastating malignant disease, PC is the fourth leading cause of cancer-related deaths in the United States, accounting for almost 7-8% of all cancer-related deaths^2,3^. In the recent years, the 5-year overall survival rate of PC has remained low, at 3%, which is partly because more than half of the PC patients are diagnosed at an advanced stage^3,4^.

Clinically, multiple parameters are used to predict the prognosis of PC, such as serum carbohydrate antigen 19–9 (CA19–9) level, tumour position, tumour size, metastatic lymph nodes, differentiation degree of the tumour, surgical margin, and adjuvant chemotherapy. It has been reported that about 80% of patients diagnosed with PC have high serum CA19-9 levels when diagnosed with PC, and the serum CA19-9 level is an independent prognostic factor for PC survival^5,6^. Tomlinson *et al*. showed that tumour grade is a strong prognostic factor of PC, and they reported a novel TNMG staging system to improve prognostic prediction in PC patients^7,8^. In addition, treatment strategies also significantly influence the prognosis of PC patients. Undoubtedly, patients with a clear surgical margin had much better survival than did those with R1 or R2 resection^9^. Further, patients who received surgery with a curative intent had better survival than did those who did not receive surgery^10,11^. Additionally, although pancreatic tumours seem to be more insensitive to chemotherapy than other solid tumours, adjuvant combination chemotherapy did yield significant benefits and lead to increased survival in PC patients, especially those with locally advanced or metastatic PC^12^.

Regarding the age at diagnosis, elderly patients, especially those aged >70 years, were reported to have a higher mortality risk than younger patients^9^. The EUROCARE-5 project, which collected data from 29 European countries, reported a poor prognosis for PC patients: one-year survival rate, 26% and five-year survival rate, 7%^13^. Moreover, the project findings showed that the relative survival reached 23% in the 15–44-year age group, which is significantly higher than that for patients aged >44 years^13^. Additionally, the data of patients aged >60 years from six European Latin countries were analysed in a sub-analysis of the EUROCARE-5 project, and the results showed that younger PC patients had a better survival^14^. However, the study did not provide data on the survival of PC patients aged <60 years. Another study analysed the data of PC patients from California and found that age >65 years was related to a slightly worse prognosis than age ≤65 years at diagnosis^15^. In all of these studies, however, the role of age at diagnosis in PC survival was not intensively discussed or stratified by multiple factors.

To investigate the association between age at diagnosis and prognosis of PC, we performed a population-based study by analysing the latest data from the Surveillance, Epidemiology, and End Results (SEER) database. Multiple confounders, including race, sex, year of diagnosis, pathological grade, American Joint Committee on Cancer (AJCC) stage, historic stage, and tumour location, were taken into consideration while calculating the PC-specific survival (PCSS). We concluded that age at diagnosis was a negative prognostic factor for PC.

## Methods

### Data source

The data for this retrospective study were downloaded from the SEER database using the official tool SEERStat provided by the website. Initially, the records of 233,786 PC patients diagnosed with PC between 1973 and 2015 were selected from the database. To analyse the data in recent years and increase the statistical power of the study, data screening was performed per the following criteria: all patients aged between 20 and 100 years and staged according to the sixth edition of the AJCC staging system. Thus, our study was limited to patients diagnosed with PC between 2004 and 2015, as the AJCC staging manual was published in 2004. Finally, the records of 126,066 PC patients with available survival time data were found eligible for the study.

To specifically analyse the correlation between age at diagnosis and prognosis of PC, the patients were divided into four groups according to their age at diagnosis: 20–40 years, 40–60 years, 60–80 years, and >80 years. There were three groups of races, including white, black, and others (American Indian/AK Native, and Asian/Pacific Islander). To assess the effect of time of diagnosis on survival, time of diagnosis was classified into three groups. Regarding the pathological grades, grades I and II, which were relatively well differentiated, formed one group, whereas grades III and IV formed the other group. Stages I and II were considered early stages of the disease, whereas stages III and IV were considered advanced stages. Historic stage in the SEER database included three categories, localized, regional, and distant. The database clearly recorded the tumour location within the pancreas.

### Statistical analyses

The endpoint of this study was PCSS, which was recorded in the database as dead (attributable to this cancer). The other causes of death were censored. The chi-square test was used to compare the distribution of baseline characteristics (race, sex, year of diagnosis, pathological grade, AJCC stage, historic stage, and tumour location) among the four age groups. In univariate analysis, the Kaplan-Meier method and log-rank test were used to compare the effects of baseline characteristics on PCSS. Significant variables in the univariate analysis were then analysed by the Cox proportional hazards model using a stepwise method. A P value <0.05 was considered statistically significant. All statistical analyses were performed with SAS 9.4, and the survival curves were constructed using GraphPad Prism 8.

## Results

### Patient characteristics

A total of 126,066 PC patients were enrolled in this population-based study. Among them, 1,422 patients were aged between 20 and 40 years, 25,692 patients were aged between 40 and 60 years, and 31,509 patients were aged >80 years. More than half of the patients were diagnosed in their 60 s and 80 s (67,443, 54.9%), and the median age at diagnosis was 70 years. The characteristics of the patients in the different age groups are shown in Table 1. Most patients diagnosed with PC were white, with the frequency of diagnosis being much higher than that among other races. Except for that among patients >80 years, the distributional difference between men and women was not high.

**Table 1:** Characteristics of pancreatic cancer patients in SEER database.

Characteristics	No.	Age groups				P value
		20–40	40–60	60–80	Over 80	
**Race**^a^						<0.0001
White	101176	1030 (73.0)	19492 (76.2)	54260 (80.6)	26394 (83.9)	
Black	15011	207 (14.7)	4202 (16.4)	7970 (11.8)	2632 (8.4)	
Other	9585	175 (12.4)	1904 (7.4)	5070 (7.5)	2436 (7.7)	
**Gender**						<0.0001
Male	63615	709 (49.9)	14757 (57.4)	35461 (52.6)	12688 (40.3)	
Female	62451	713 (50.1)	10935 (42.6)	31982 (47.4)	18821 (59.7)	
**Year of diagnosis**						<0.0001
2004–2007	36900	402 (28.3)	7788 (30.3)	19101 (28.3)	9609 (30.5)	
2008–2011	42084	449 (31.6)	8598 (33.5)	22311 (33.1)	10726 (34.0)	
2012–2015	47082	571 (40.2)	9306 (36.2)	26031 (38.6)	11174 (35.5)	
**Pathological grade**						<0.0001
Grade I/II	22925	512 (36.0)	5939 (23.1)	13322 (19.8)	3152 (10.0)	
Grade III/IV	16804	170 (12.0)	3957 (15.4)	10024 (14.9)	2653 (8.4)	
Unknown	86337	740 (52.0)	15796 (61.5)	44097 (65.4)	25704 (81.6)	
**AJCC stage**						<0.0001
I/II	37023	457 (32.1)	7346 (28.6)	20614 (30.6)	8606 (27.3)	
III/IV	69449	667 (46.9)	15451 (60.1)	38741 (57.4)	14590 (46.3)	
Unknown	19594	298 (21.0)	2895 (11.3)	8088 (12.0)	8313 (26.4)	
**Historic stage**^c^						<0.0001
Localized	13111	332 (23.4)	2242 (8.7)	6168 (9.2)	4369 (13.9)	
Regional	35604	333 (23.4)	7614 (29.6)	20496 (30.4)	7161 (22.7)	
Distant	65938	702 (49.4)	14728 (57.3)	36554 (54.2)	13954 (44.3)	
Unstaged	11413	55 (3.9)	1108 (4.3)	4225 (6.3)	6025 (19.1)	
**Tumor location**^b^						<0.0001
Head	59624	556 (39.1)	11998 (46.7)	32569 (48.3)	14501 (46)	
Body	14645	139 (9.8)	3202 (12.5)	8183 (12.1)	3121 (9.9)	
Tail	16592	314 (22.1)	4003 (15.6)	9179 (13.6)	3096 (9.8)	
Overlapping	8852	114 (8)	1993 (7.8)	4942 (7.3)	1803 (5.7)	
Other	26353	299 (21)	4496 (17.5)	12570 (18.6)	8988 (28.5)	

Data are displayed as n (%). a: There were 294 missing values in the variable of Race. ‘other’ means American Indian/AK Native, and Asian/Pacific Islander. b: tumor location means the specific location of tumor in the pancreas. c: Using SEER historic stage A. For detailed description of SEER historic stage A, please refer to the website: https://seer.cancer.gov/tools/ssm/SSM2018-DIGESTIVE-AND-HEPATOBILIARY-SYSTEMS.pdf.

As shown in Table 1, new cases of PC increased in all age groups between 2004 and 2015. According to the sixth AJCC staging system, the patients were preferentially diagnosed with stage III/IV PC at their first diagnosis. According to the historic staging criteria of SEER, approximately half of the patients had distant organ involvement, regardless of the age at PC diagnosis. In addition, 47.3% of the pancreatic tumours were located in the head of the pancreas at diagnosis, which is three times higher than the proportion of tumours located in the pancreatic body or tail. This trend was observed in all age groups.

### Comparisons of the survival curves

**Figure 1:**
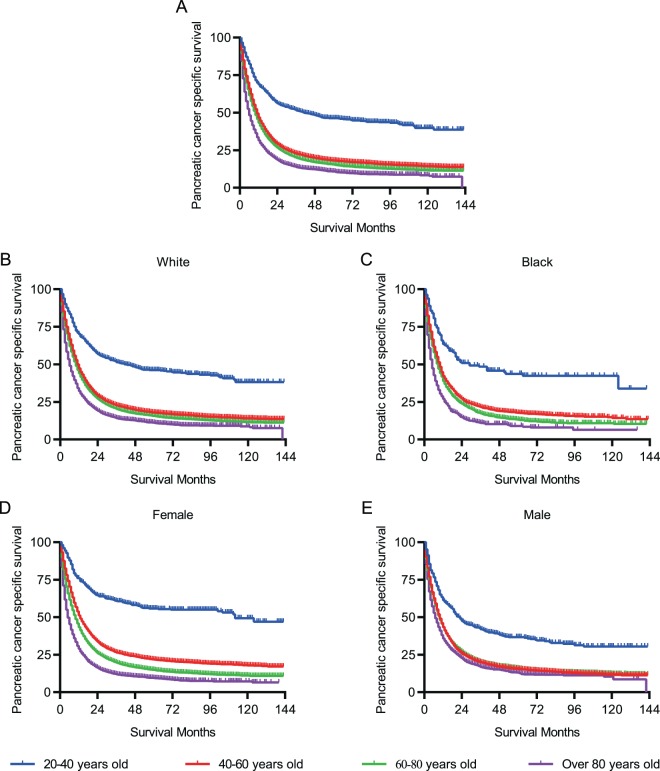
Pancreatic cancer-specific survival of patients. (**A**) Survival curves in different age groups. (**B,C**), Survival curves stratified by race. (**D,E**) Survival curves stratified by gender.

PCSS was calculated to assess the survival of patients with PC. The median PCSS in each age group was 36.0, 10.0, 8.0, and 4.0 months, respectively. The five-year PCSS of patients aged 20–40 years was about 1.5 times higher than that of patients aged 40–60 years and 2 times higher than that of patients aged 60–80 years (Table 2 and Fig. 1A), indicating that the frequency of PCSS reduced with increasing age. As is shown in Fig. 1B,C, race did not affect the trend of low PCSS with age. A prominent trend was that PCSS decreased with age in women, whereas male patients tended to show PCSS when they were aged >40 years (Fig. 1D,E). The five-year PCSS in female patients was 54.4%, 20.7%, 13.4, and 7.5% in the four age groups, respectively, whereas in male patients, the PCSS was 34.6%, 14.1%, 14.1%, and 10.7%, respectively.

**Table 2:** Univariate and multivariate survival analysis for pancreatic cancer specific survival in SEER database.

Characteristics	Median survival time (months)	5-year PCSS (%)	Univariate analysis		Multivariate analysis	
			Log rank χ^2^ test	P value	HR (95% CI)	P value
**Race**			46.42	<0.0001		
White	8.0	13.9			Reference	
Black	7.0	13.0			1.11 (1.09–1.13)	<0.0001
Other	7.0	14.5			/	/
**Gender**			2.0	0.1590		
Male	8.0	13.8			/	/
Female	7.0	14.0			/	/
**Year of diagnosis**			849.6	<0.0001		
2004–2007	6.0	11.1			Reference	
2008–2011	7.0	13.6			0.90 (0.89–0.92)	<0.0001
2012–2015	9.0	/			0.81 (0.80–0.83)	<0.0001
**Pathological Grade**			9351.5	<0.0001		
Grade I/II	22.0	31.1			Reference	
Grade III/IV	9.0	11.3			1.65 (1.61–1.70)	<0.0001
Unknown	5.0	8.8			1.78 (1.75–1.82)	0.0003
**AJCC stage**			13336.0	<0.0001		
I/II	17.0	24.8			Reference	
III/IV	4.0	4.6			1.52 (1.48–1.56)	<0.0001
Unknown	7.0	22.1			0.94 (0.90–0.97)	<0.0001
**Historic stage**			14466.2	<0.0001		
Localized	26.0	42.2			Reference	
Regional	14.0	17.2			1.37 (1.32–1.41)	<0.0001
Distant	4.0	5.9			2.00 (1.93–2.08)	<0.0001
Unstaged	4.0	11.3			2.14 (2.05–2.24)	<0.0001
**Tumor location**			2090.1	<0.0001		
Head	9.0	13.5			Reference	
Body	8.0	13.8			0.92 (0.90–0.94)	<0.0001
Tail	7.0	21.4			0.92 (0.90–0.94)	<0.0001
Overlapping	7.0	11.7			/	/
Other	4.0	10.8			1.11 (1.09–1.13)	<0.0001
**Age**			3967.2	<0.0001		
20–40	36.0	44.7			Reference	
40–60	10.0	16.9			1.86 (1.72–2.01)	<0.0001
60–80	8.0	13.8			2.22 (2.05–2.40)	<0.0001
Over 80	4.0	8.7			3.30 (3.05–3.57)	<0.0001

Abbreviations: PCSS, pancreatic cancer specific survival; HR, hazard ratio; CI, confidence interval.

**Figure 2:**
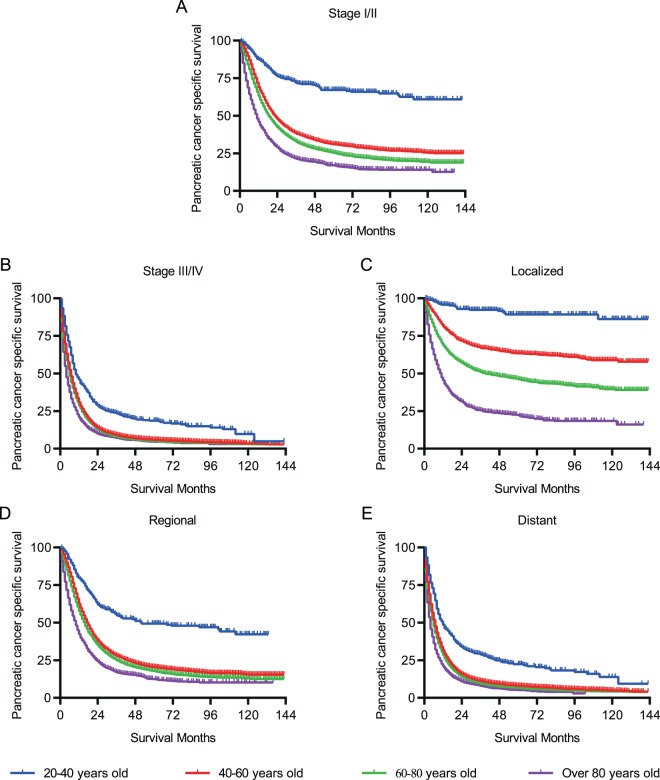
Pancreatic cancer-specific survival of patients at diverse stages. (**A,B**) Survival curves stratified by AJCC stage. (**C–E**) Survival curves stratified by historic stage in SEER database.

**Figure 3:**
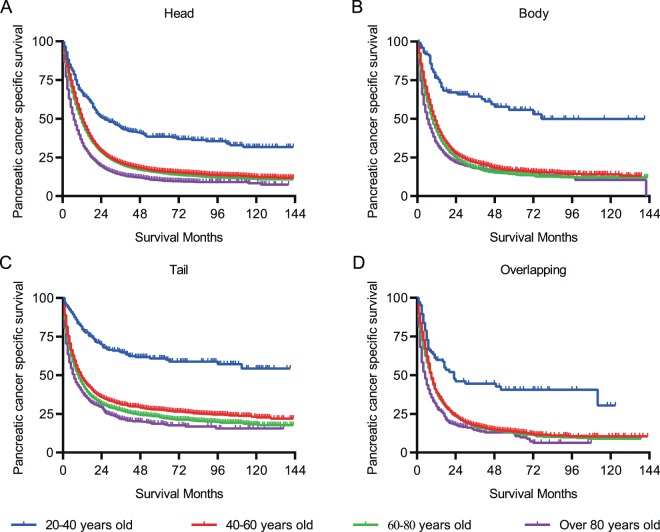
Survival curves stratified by specific tumor location in the pancreas.

**Figure 4:**
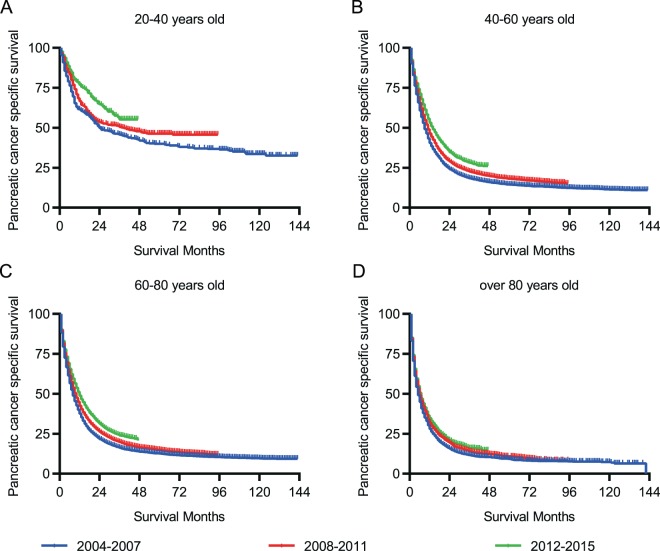
Survival curves stratified by year of diagnosis of pancreatic cancer.

To reduce the bias of various stages of PC on PCSS, we stratified PC patients of different groups according to the AJCC staging system and SEER historic staging system. In stage I/II PC patients, the PCSS decreased with age, whereas in more advanced PC cases (stage III/IV), the PCSS was almost parallel in patients >40 years and those aged between 20 and 40 years (Fig. 2A,B). Similarly, in patients with a localized tumour, age was associated with a drastic decline in PCSS (Fig. 2C). Nevertheless, no marked difference in age-related survival decline could be observed in patients >40 years when regional or distant organs were involved (Fig. 2D,E). Moreover, specific tumour location in the pancreas was also taken into consideration to assess the PCSS. Likewise, patients <40 years had a much better survival than those of other age groups despite the location of the pancreatic tumours (Fig. 3). Additionally, year of diagnosis was also analysed as a stratifying factor. In the 20–40 years group, the median survival times for PC diagnosed in 2004–2007 and 2008–2011 were 24.0 and 39.0 months, respectively (Fig. 4A), whereas the median survival time was >39.0 months for patients diagnosed between 2012 and 2015 (without a specific number because of incomplete follow-up). However, for patients aged >40 years, the negligible survival improvement did not reach any clinical significance, although the survival curves were statistically significant (Fig. 4B–D).

### Results of univariate and multivariate analyses

In the univariate analysis, stratification factors, such as race, sex, year of diagnosis, pathological grade, AJCC stage, historic stage, tumour location, and age were used to evaluate PCSS and calculate the five-year PCSS. All of these factors, except sex, were significantly associated with PCSS (Table 2).

In multivariate analysis, all significant stratification factors were included in the Cox model (Table 2). Race and sex were not found to be prognostically important for assessing the survival of PC patients. Additionally, recent diagnosis of PC was found to be associated with a better survival than diagnosis in previous years. Undoubtedly, PC patients with tumours of higher grades had a higher risk of death than did those with tumours of pathological grade I/II. Likewise, advanced PC patients in stage III/IV or with distant organ involvement had a much poorer prognosis than did those outside this grouping. Compared with tumours in the head, tumours localized in the body and tail of the pancreas appeared to be associated with a favourable prognosis. Finally, the mortality risk of PC patients aged between 40 and 80 years was twice that of the patients aged below 40 years. However, patients aged >80 years had a mortality risk three times higher than that of patients aged <40 years. Therefore, age was an independent factor for predicting the prognosis of PC patients.

## Discussion

In all cancers, age is an important indicator of prognosis. Per the data obtained from the official website of Cancer Research UK, the mortality rate is strongly correlated with age for all types of cancers, with specifically high mortality rates for elderly patients. Patients aged between 15 and 40 years have the highest cancer survival rate, whereas those aged between 80 and 99 years have the lowest cancer survival rate^16^.

In this study, we focused on the influence of age at diagnosis on the prognosis of PC, and age at diagnosis was simply divided into four groups to comprehensively analyse the prognosis at different ages. Additionally, the data were stratified by race, sex, year of diagnosis, pathological grade, AJCC stage, historic stage, and tumour location to assess the effects of age at diagnosis on PC survival. It has been demonstrated from our results that PC patients aged between 20 and 40 years had the best cancer survival, especially those with a lower pathological grade and diagnosed at an early stage. Consistent with previous reports^4^, no significant difference in cancer survival was found between men and women. However, our results revealed a more prominent descending trend in the 5-year PCSS among women than among men. A study analysing the data of the National Center for Health Statistics (NCHS) from 1980 to 2014 reported that the 5-year relative survival of PC patients had increased to 8%^17^, which was in accordance with our results. Pathological grade, as a measure of the degree of differentiation of tumour, has been consistently found to be an independent prognostic factor of the overall survival after pancreatic tumour resection^7,18–20^. In this study, our results showed that patients with a higher tumour grade had a much worse prognosis than did those with lower tumour grades. Most PC patients were diagnosed at a relatively advanced stage, and clinical staging has been accepted as a prognostic factor for cancer treatment. However, the effect of age at different stages on the survival rate has not been reported^20^. Our results showed that younger patients diagnosed at an early stage had the best survival, and the survival rate declined with increasing age. However, the survival rate was similar in patients who were diagnosed at the age of 40 years or older. Although some studies reported that tumours in the body and tail of the pancreas showed more aggressive behaviour, they had a small sample size^21,22^.

Most of the previous studies involved a limited number of patients. In this large sample-sized study, patients with tumours in the tail of the pancreas had the best survival and lowest risk of death. On further adjusting for the potential baseline confounding factors, a Cox regression model was constructed to assess the effect of each clinicopathological characteristic on the survival rate. As expected, age at diagnosis was strongly correlated with the risk of death, and patients older than 80 years had the worst survival. In one study, among the PC patients who underwent extended pancreatectomy, although age was not associated with in-hospital mortality, the survival of patients older than 70 years was worse than that of the younger patients^9^, consistent with our results. In the Cox model, a 20% of mortality risk was found among patients diagnosed between 2012 and 2015 compared with those diagnosed in 2004–2007. This might be because of the improvement in medical treatment, such as modified surgical procedures, improved chemotherapeutic regimens, and emerging immunotherapy treatments. Besides, our stratified analysis revealed that patients younger than 40 years benefited the most in recent years. In some retrospective studies, younger patients tended to receive more aggressive treatment than elderly patients^10^, which could be a reason why younger patients showed better survival.

Apart from the factors discussed above, many other factors also influence the survival of PC patients. Surgical treatment of resectable pancreatic tumour remains the main curative option for this cancer. A positive surgical margin significantly correlates with a higher local recurrence and a lower overall survival^23–25^. However, because of the limitations of the databases, the margin status was not available for analysis. Although pancreatic tumours are insensitive to pharmacological treatment because of complex molecular mechanisms and heterogeneity of the tumour cells^26^, certain improvements in chemotherapy, targeted therapy, and immunotherapy have been made^27,28^. For locally advanced pancreatic cancer, FOLFIRINOX (leucovorin and fluorouracil plus irinotecan and oxaliplatin) was reported to increase the pooled median overall survival to 24.2 months, longer than the 15.0 months for patients treated with gemcitabine^29^. Unfortunately, no detailed treatment strategies are available in the database and this prevented us from analysing the combination of age and different treatment strategies.

In recent years, the number of new PC cases is increasing, probably because of the use of advanced screening methods and popularization of regular physical examination. Unquestionably, novel treatment strategies for PC play a significant role in improving the survival rate, but much more needs to be done to augment the extremely low five-year survival rate. Despite the poor survival of PC, this study showed disparate survival rates among patients in different age groups. Moreover, considering the age at diagnosis as a negative prognostic factor might help clinicians make a more precise prognostication for PC patients. Although this study was based on a large sample size, certain confounders remained unaddressed in this study because of the limitations of the SEER database.

## Data Availability

All data analyzed in this article were available on the official website of SEER (https://seer.cancer.gov).
